# Growing Tibetan Pigs Adapt to High-Fiber Diets by Enhancing Fiber Degradation Capacity

**DOI:** 10.3390/vetsci12101010

**Published:** 2025-10-18

**Authors:** Zhima Lamu, Shuyu Hao, Boxuan Li, Sichen Yang, Zhenda Shang, Peng Shang, Suozhu Liu, Yan Lin, Zhankun Tan

**Affiliations:** 1 College of Animal Science, Xizang Agricultural and Animal Husbandry University, Linzhi 860000, China; 15183623416@163.com (Z.L.); haoshuyu0218@163.com (S.H.); 13869230603@163.com (B.L.); 15531692263@163.com (S.Y.); shangzhenda1988@163.com (Z.S.); nemoshpmh@126.com (P.S.); liusuozhu@xza.edu.cn (S.L.); 2 National Center for International Research on Animal Gut Nutrition, National Experimental Teaching Demonstration Center of Animal Science, Nanjing Agricultural University, Nanjing 210095, China; linyan@njau.edu.cn

**Keywords:** Tibetan pig, dietary high fiber, intestinal microorganism, fiber degradation capacity

## Abstract

**Simple Summary:**

The Tibetan pig is a unique breed native to the Qinghai–Tibet Plateau, which has adapted to the grazing system of high-altitude pastures and exhibits remarkable tolerance to crude fiber. However, with the transition to large-scale intensive farming, their diets have been optimized for short-term growth efficiency. This nutritional composition conflicts with the Tibetan pig’s evolved adaptation to high-fiber intake. Despite possessing high fiber tolerance, the mechanisms by which enzymes and bacteria synergistically enable efficient utilization of coarse fiber in the Tibetan pig gut remain unclear. This study examined the effect of high-fiber diets on the proliferation of fecal fiber-degrading bacteria, activity of fiber-digesting enzymes, and production of short-chain fatty acids in Tibetan pigs during their growth phase.

**Abstract:**

The systematic analysis of the synergistic mechanism between microbial fiber-degrading enzymes and short-chain fatty acids under high-fiber diet conditions is limited. In this study, we evaluated the effects of a high-fiber diet on the growth performance, nutrient digestibility, blood and serum metrics, cellulase/hemicellulase activity, and fecal microbial composition of growing Tibetan pigs. Forty Tibetan pigs were allocated to a control group (CON, the diet contains 5% crude fiber) or a high-fiber group (HF, the diet contains 10% crude fiber) based on crude fiber levels as a blocking factor. The pre-trial period was 7 d, and the formal trial lasted 28 d. CON group and HF group showed no effect on growth performance and nutrient apparent digestibility (*p* > 0.05). The HF group showed significantly higher fecal cellulase and hemicellulase activities than those of the CON group (*p* < 0.05). Additionally, the HF group showed significantly elevated levels of acetic, propionic, and butyric acids, as well as increased relative abundances of *Fibrobacter* and *p-75-a5* in the feces (*p* < 0.05). The correlation analysis revealed that *Fibrobacter* exhibited significant positive correlations with acetic acid, butyric acid, cellulase, and hemicellulase, whereas *p-75-a5* was significantly positively correlated with hemicellulase (*p* < 0.05). In conclusion, this study provides strong evidence that the efficient utilization of dietary fiber by Tibetan pigs results from highly specialized microbial mechanisms in their large intestine, as reflected by their fecal microbiota composition. *Fibrobacter* and *p-75-a5* play a crucial role in enabling these pigs to utilize fiber effectively. Certain specific microbiota secrete a greater quantity of enzymes to facilitate the decomposition of dietary fiber, and this process ultimately leads to the generation of more metabolites.

## 1. Introduction

Tibetan pigs have undergone millennia of natural selection across various terrains of the Tibetan Plateau, leading to their exceptional adaptation to harsh environmental conditions [1]. As a distinct genetic resource in this region, they exhibit specialized physiological mechanisms that enable them to thrive in high-altitude, low-oxygen environments [2]. Tibetan pigs exhibit robust fiber digestion capabilities [3], providing a crucial physiological foundation for their adaptation to various diets and the effective utilization of crude fiber [4,5]. The focus of animal husbandry research has recently shifted towards improving nutrient utilization efficiency in stall-feeding systems [6]. An important consideration in this domain is the optimization of crude fiber levels to ensure the healthy growth of Tibetan pigs [7]. With concentrated feed prices, raw materials with high crude fiber are relatively inexpensive, and increasing the proportion of crude fiber in diets can effectively reduce breeding costs [8]. Thus, optimizing the dietary fiber levels is essential for balancing the healthy growth of Tibetan pigs with cost control.

Fiber, a prolific and globally distributed renewable resource, forms the primary structure of plant cell walls and is mainly composed of non-starch polysaccharides and lignin [9,10]. It is an effective alternative to growth promoters and positively influences the growth and health of monogastric animals [11]. Fermented fiber feed enhances growth by increasing appetite and feed intake, thereby contributing to the widespread use of fiber-rich materials in pig diets [12,13]. Adding dietary fiber to Tibetan pig feed not only matches the high-fiber dietary characteristics of their original habitat, but also improves feed conversion efficiency and average daily gain by shaping a unique intestinal microbiota to help Tibetan pigs enhance their buffering adaptability to high-fiber diets, and it also increases the satiety of Tibetan pigs while enabling the efficient and stable degradation of fiber by their microorganisms to maintain a certain fiber digestibility [14,15]. Additionally, dietary fiber regulates appetite, improves blood glucose and lipid responses, and modulates plasma cholesterol by restricting bile salt absorption, enhancing digestive function, and optimizing nutrient digestion and absorption [16,17]. Thus, dietary fiber is vital for Tibetan pig nutrition.

The gastrointestinal tract of animals hosts trillions of microorganisms which are crucial for host health and perform essential metabolic, immune, and protective functions [18]. Gut health can be promoted by dietary fiber through modulating the composition and metabolism of the gut microbiota, stimulating mucus production, enhancing gut motility, and maintaining gut barrier integrity [19]. Intestinal microbiota are crucial for the digestive system of Tibetan pigs because their intestinal tract contains a diverse community of microorganisms, including *Fibrobacter*, *Alloprevotella*, and *Succinivibrio*. These bacteria efficiently degrade cellulose, hemicellulose, and other intricate polysaccharides, thereby enhancing the digestion and assimilation of high-fiber diets in Tibetan pigs [20]. Intestinal microorganisms are involved in the synthesis of short-chain fatty acids (SCFAs), multivitamins, and amino acids, thereby providing supplementary energy sources for Tibetan pigs and enhancing their resilience to the harsh plateau conditions [21]. The gut microbiota produce SCFAs through the fermentation of dietary fiber, which are an energy source for the colonic epithelium and also lower luminal pH, thereby inhibiting the overgrowth of pathogenic bacteria [22,23]. SCFAs are important regulators of proliferation, immunity, and metabolism in pigs [24,25,26,27]. Consequently, a fiber-rich diet positively affects the gut microbiota, as well as the host’s metabolic, immune, and gastrointestinal functions [28,29]. However, current studies have two main limitations. First, there has been a predominant focus on describing the microbial bacteria’s composition, with limited systematic analysis of the synergistic mechanism between microbial fiber-degrading enzymes and SCFAs under high-fiber diet conditions. Additionally, the pathways through which core functional bacteria, such as *Fibrobacter*, regulate metabolites via enzyme activity remain insufficiently understood. Second, existing studies on the adaptation of Tibetan pigs to high-fiber diets during the growth stage have primarily concentrated on individual indices, such as microbial composition or digestibility, without establishing a comprehensive regulatory chain comprising dietary fiber levels, changes in microbial communities, enzyme activities, and SCFA production. These limitations affect the elucidation of efficient adaptation mechanisms of Tibetan pigs to high-fiber diets in plateau environments. Addressing these gaps provides opportunities for further exploration.

We hypothesized that increasing the crude fiber content in the diet of maturing Tibetan pigs could enhance the production of short-chain fatty acids by facilitating the proliferation of intestinal fiber-degrading bacterial genera and increasing the activity of fiber-digesting enzymes. This process would establish a synergistic relationship among bacteria, enzymes, and metabolites, facilitating the adaptation of Tibetan pigs to a high-fiber diet. Two diet groups were established: one with the diet contains 5% crude fiber (CON) and the other with the diet contains 10% crude fiber (HF). This study investigated the effects of feeding high fiber diet on growth performance, nutrient digestibility, blood parameters, fecal microbiota, enzyme activity and metabolites in Tibetan pigs.

## 2. Materials and Methods

The procedures used in this study were approved by the Institutional Animal Care and Use Committee of Xizang Agricultural and Animal Husbandry University (Approval No.: XZA-2023-003). The corn, lignocellulose, and soybean hulls in the dietary fiber were sourced from Linzhi Bajian Tibetan Pig Industry Feed Processing and Production Co., Ltd., Xizang Autonomous Region, China.

### 2.1. Experimental Design, Diet, and Animal Housing

Forty Tibetan pigs (average body weight 21.52 ± 3.45 kg) at 120 d of age were randomly divided into two groups, each set comprised 20 repetitions, with an equal number of males and females, and each repetition contained one pig: (1) a control group (CON, the diet contains 5% crude fiber) and (2) a high-fiber group (HF, the diet contains 10% crude fiber). The experimental period lasted for 28 days following a pre-experimental period of 7 days. According to the nutritional requirements of GB/T39235-2020 standard and with reference to the “Chinese Fat-Type Growth-Finishing Pig Feeding Standards” [30,31]. Two diets were fed with equal energy and nitrogen but with different fiber levels (Table 1). The test pigs were kept in separate pens (1.5 m × 2.0 m). Each pen was equipped with one stainless steel trough feeder placed on the left side and one automatic nipple drinker on the right side, feed and water were provided ad libitum. The pen floor was made of reinforced concrete, and covered with a 5 cm thick layer of clean rice husk bedding (replaced every 3 days) to maintain dryness and comfort. The rearing environment was controlled at a temperature of 16–21 °C and humidity of 55–65%. During the experimental period, the pens were cleaned daily to remove feces and leftover feed. Breeders conducted daily health monitoring, recording indicators such as feed intake, fecal consistency, and mental state; any pigs with abnormal symptoms were immediately isolated and examined.

### 2.2. Growth Performance Evaluation

Individual body weight (BW) and feed intake of pigs were monitored every 2 weeks, and average daily gain (ADG), average daily feed intake (ADFI), and feed-to-weight ratio (F:G) were calculated. The average daily feed intake (g/d) was calculated as follows: (total feed weight during the trial period–remaining feed weight during the trial period)/number of days in the trial. The average daily weight gain (g/d) was calculated as follows: (final body weight–initial body weight)/number of days in the trial. The feed-to-weight ratio was determined by dividing the average daily feed intake by the average daily weight gain [32].

### 2.3. Sample Collection

After the 28-day experiment, following a 12 h fast, 20 pigs (10 female and 10 male per treatment) were selected per group, and blood samples were collected from jugular veins. The blood was divided into two portions: One portion was collected in a procoagulant vacuum tube and centrifuged at 3000 rpm for 15 min to collect the serum, which was then transferred to 1.5 mL centrifuge tubes for multiple serum indicator testing. The remaining portion was collected directly into ethylenediaminetetraacetic acid dipotassium salt (EDTA-K_2_) anticoagulant tubes. Both samples were stored in –20 °C freezers. Fecal samples were collected between days 21 and 24, with a total of four collections. When the pig was defecating or about to defecate, rectal stimulation was performed. One person promptly collected the feces using a self-sealing plastic bag to prevent contact with the ground, while another person marked the pig with spray lacquer. This fecal collection method allowed for clear tracing of fecal samples to their corresponding pig IDs and ensured proper organization of each pig’s fecal samples. Prior to sampling, tweezers, 2 mL cryopreservation tubes, and gloves used for microbial sample collection were autoclaved and dried to ensure sterility. Uncontaminated fecal samples from the central portion were collected into sterile tubes: portions were stored at −80 °C for 16S rRNA sequencing, fiber-degrading enzyme activity determination, and SCFA analysis, while the remaining feces were stored at −20 °C for chemical analysis.

### 2.4. Apparent Total Tract Nutrient Digestibility Analysis

The feed and fecal samples used for nutrient digestibility analysis in this section were collected and processed in chapter 2.3, and were subsequently dried, ground, and sieved through a 1 mm sieve. Dry matter (DM) was analyzed using Method 934.01, crude protein (CP) using Method 976.05, ether extract (EE) using Method 922.06, and crude fiber (CF) was determined, all following the guidelines of the Association of Official Analytical Chemists (AOAC, 2007). Acid-insoluble ash (AIA) was used as an marker (indigestible and unabsorbed in the gastrointestinal tract) to calculate digestibility. Its content in both feed and fecal samples was determined with reference to AOAC Official Method 942.05 [33]. Digestibility was determined using the following formula: apparent total tract digestibility (ATTD) of nutrients = [1 − (acid-insoluble ash content in feed/acid-insoluble ash content in feces) × (nutrient content in feces/nutrient content in feed)] × 100%.

### 2.5. Blood Parameter Analysis

Blood samples for hematological examination were collected into tubes containing EDTA-K_2_ to prevent coagulation. After gentle inversion to ensure homogeneity, the samples were analyzed immediately following the operational procedures outlined in the automated hematology analyzer’s instruction manual to avoid cellular degradation. All hematological parameters were measured using an automated hematology analyzer (Dymind-DH71CRP, Shenzhen, China). The parameters assessed included white blood cells (WBC), red blood cells (RBC), hemoglobin (HGB), hematocrit (HCT), platelets (PLT), and procalcitonin (PCT) levels; calculated parameters, including mean corpuscular volume (MCV), mean corpuscular hemoglobin content (MCH), mean corpuscular hemoglobin concentration (MCHC), mean platelet volume (MPV), platelet distribution width (PDW), and red blood cell distribution width (RDW), were all derived from the above directly measured values or cell volume distribution data detected by the analyzer. All hematological analyses were performed within two hours of sample collection to prevent artifacts and ensure data accuracy.

### 2.6. Serum Parameter Analysis

Serum parameters were analyzed using a biochemical analyzer (Toshiba-BS120, Beijing, China) with kits produced by Suzhou Keming Biotechnology Co., Ltd. (Suzhou, China); the specific operating procedures were carried out with reference to the kit instructions, including alanine aminotransferase (ALT), aspartate aminotransferase (AST), total protein (TP), albumin (ALB), globulin (GLO), serum alkaline phosphatase (ALP), urea (UREA), creatinine (CREA), total cholesterol (TC), and blood glucose (GLU); The AST/ALT ratio and albumin/globulin ratio (A/G) were calculated from the measured values.

### 2.7. Enzyme Activity Analysis

Cellulase and hemicellulase activities in fecal supernatants of experimental animals were measured using porcine. Cellulase and hemicellulase ELISA kits (Jiangsu Enzyme Exemption Industry Co., Ltd., Nanjing, China) following manufacturer’s instructions. Cellulase standards were diluted to 32, 16, 8, 4, 2 IU/L by adding 15 μL original standard to 150 μL diluent. Blank, standard, and sample wells were set: 50 μL standard per standard well; 40 μL diluent + 10 μL sample (5-fold dilution) per sample well. Samples were added to well bottoms and mixed gently. Plates were sealed and incubated at 37 °C for 30 min. After 5 washes (30-fold diluted washing solution, 30 s each), 50 μL enzyme reagent (except blanks) was added, followed by incubation and washing. Then 50 μL chromogens A and B were added, incubated at 37 °C in dark for 10 min. Reaction was terminated with 50 μL stop solution (blue to yellow). OD values were measured using a microplate reader (Thermo VARIOSKAN LUX, Shanghai Yetuo Technology Co., Ltd., Shanghai, China) at 450 nm within 15 min, using blank well as reference.

### 2.8. Analysis of Fecal Short-Chain Fatty Acids

Each fecal sample (0.2 g) was mixed with 0.8 mL of 0.1 mol/L hydrochloric acid and thoroughly vortexed for 1 min to ensure complete homogenization, then incubated on ice for 30 min. The mixture was centrifuged at 12,000 rpm for 15 min in a Centrifuge 5804 (Eppendorf, Hamburg, Germany). The supernatant was filtered through a 0.22 μm membrane and analyzed for SCFA content using a gas chromatograph (SCION 456i, Shanghai, China).

### 2.9. Fecal Microbiota Diversity Analysis

Fecal samples were collected from groups consisting of ten repetitions (one pig per repetition), including 5 males and 5 females, to balance gender and avoid gender-related biases. were analyzed for fecal microbiota diversity at Shanghai Personalbio Biotechnology Co., Ltd. The primer sequences were as follows: forward primer, F: ACTCCTACGGGAGGCAGCA; reverse primer, R: GGACTACHVGGGTWTCTAAT. Total microbial DNA was extracted from fecal samples using a QIAamp Fast DNA Stool Mini Kit. Paired-end sequencing of the V3–V4 region of the 16S rRNA gene of fecal bacteria was conducted on the Illumina platform, and QIIME2 (2019.7) software was used to eliminate erroneous and low-quality sequences. Vsearch software (2.0.5) was employed to cluster the sequences obtained. The average read length after pruning was 419, with a minimum read length of 404. Amplicon Sequence Variables (ASVs) were categorized based on a 97% sequence similarity. Representative sequences of Operational Taxonomic Units (OTUs) were compared with the GenBank database to acquire taxonomic information. Fecal microbiota diversity was assessed using four α-diversity indices (Shannon, Simpson, Chao1, and Faith_pd), and non-metric multidimensional scaling (NMDS) analysis was conducted in Origin software. The default ASV/OTU table or the absolute abundance table of taxa at various hierarchical levels (phylum, class, order, family, and genus) was utilized. The analysis involved employing the “classify_samples_ncv” function in q2-sample-classifier to conduct random forest analysis and nested stratified cross-validation. A random forest analysis was performed to identify the top 15 marker genes at the genus level. Subsequently, a Venn diagram was used to illustrate the overlap between the top 20 genes based on their relative abundance and the top 15 genes based on their importance at the genus level. By analyzing the composition of fecal microorganisms, a correlation analysis was conducted based on Pearson’s correlation coefficient with Tibetan pig fiber indicators.

### 2.10. Statistical Analysis

Microsoft Excel 2019 was used for data entry and preliminary organization, before conducting formal statistical analysis, performed using SPSS software (version 21.0) the assumptions of normality and homogeneity of variance for continuous variables were verified: normality was tested using the Shapiro–Wilk test, and homogeneity of variance was assessed using Levene’s test. For continuous variables conforming to both the normality and homogeneity of variance assumptions, independent sample *t*-tests to compare differences between groups (each group consisted of twenty repetitions, with one pig per repetition). All significance was based on a probability threshold of *p* < 0.05. Significance levels are denoted as * *p* < 0.05, ** *p* < 0.01, and *** *p* < 0.001.

## 3. Results

### 3.1. Influence of High-Fiber Diet on Growth Performance of Tibetan Pigs

No significant difference was observed in the initial body weight of Tibetan pigs between the HF and CON groups (*p* > 0.05; Table 2), confirming the homogeneity of the two groups at the start of the experiment. Consistent with this, compared to the CON group, the HF group also showed no significant effects on the final body weight, average daily gain, average daily feed intake, or feed-to-gain ratio of Tibetan pigs at each stage (*p* > 0.05).

### 3.2. Influence of High-Fiber Diet on Apparent Nutrient Digestibility of Tibetan Pigs

No significant differences were observed in the digestibility of crude protein, ether extract, and crude fiber between the HF and CON groups (*p* > 0.05; Table 3), the amount of crude fiber digested in HF group was twice that of CON group (Formula = CF digestibility × crude fiber content; calculated as CON: 63.28% × 5% = 3.16%, HF: 63.40% × 10% = 6.34%, nearly doubling that of the CON group).

### 3.3. Influence of High-Fiber Diet on Blood Parameters of Tibetan Pigs

No significant differences were observed in the WBC, RBC, HGB, HCT, MCV, MCH, MCHC, RDW, PLT, MPV, PDW, and PCT levels between the HF and CON groups (*p* > 0.05; Table 4).

### 3.4. Influence of High-Fiber Diet on Serum Parameters of Tibetan Pigs

No significant differences were observed in the ALT, AST, TP, ALB, GLO, A/G, ALP, UREA, CREA, TC, and GlU levels between the HF and CON groups (*p* > 0.05; Table 5).

### 3.5. Influence of High-Fiber Diet on Enzyme Activity of Tibetan Pigs

Compared to the CON group, the HF group had significantly higher levels of cellulase and hemicellulase activities in Tibetan pigs (*p* < 0.05; Table 6)

### 3.6. Influence of High-Fiber Diet on SCFAs of Tibetan Pigs

The levels of acetic, propionic, and butyric acids were significantly higher in the HF group than in the CON group (*p* < 0.05). However, changes in the crude fiber levels in the Tibetan pig diet did not significantly affect the levels of formic, isobutyric, isovaleric acid, and valeric acid between the two groups (*p* > 0.05; Table 7).

### 3.7. Influence of High-Fiber Diet on Fecal Microbiota of Tibetan Pigs

**Figure 1:**
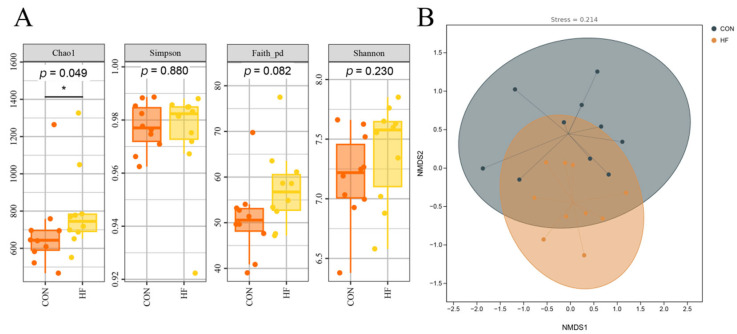
Effects of different dietary fiber levels on the α-diversity index and β-diversity of fecal microbiota in confined Tibetan pigs: (**A**) α-diversity indices between groups; (**B**) non-metric multidimensional scaling (NMDS) plot between groups, with stress value = 0.214. CON, the diet contains 5% crude fiber; HF, the diet contains 10% crude fiber; Values are the means ± merged SEM (*n* = 20). In (**A**), orange boxes represent the CON group and yellow boxes represent the HF group; in (**B**), black dots represent the CON group and orange dots represent the HF group. “*” indicates statistically significant differences in the two groups (*p* < 0.05).

The Chao1 index in the HF group was significantly higher than that in the CON group (*p* < 0.05; Figure 1A). No significant differences were observed in the Simpson, Faith_pd, and Shannon indices between the two groups (*p* > 0.05). Non-metric multidimensional scaling (NMDS) was used to analyze the β-diversity of the microbiota, and the stress value of 0.214 indicated that the ordination results were reliable. Microbiota structures in the CON and HF groups were not significantly different (Figure 1B).

**Figure 2:**
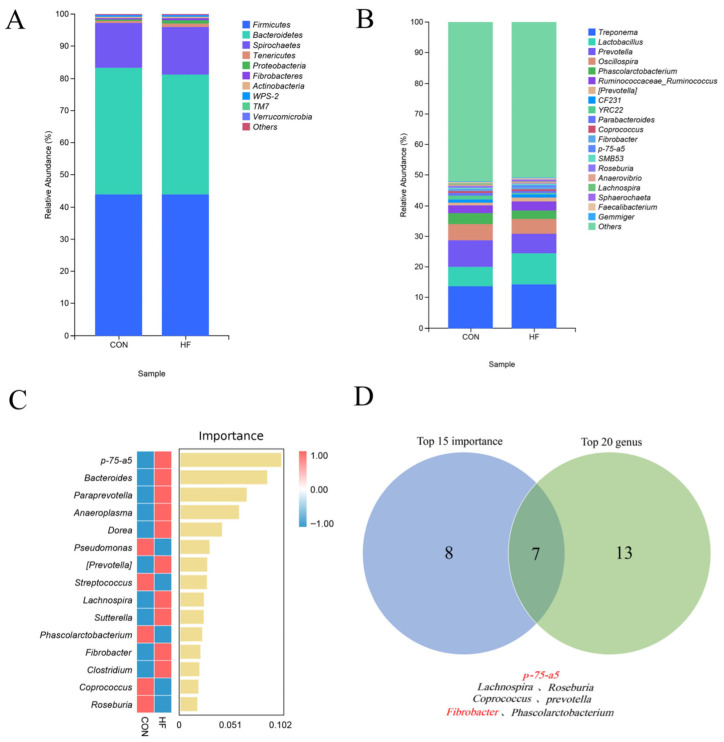
Effects of different dietary fiber levels on the relative abundance of fecal microbiota in confined Tibetan pigs: (**A**) the top 10 phyla with the highest relative abundance at the phylum level; (**B**) the top 20 genera with the highest relative abundance at the genus level; (**C**) the top 15 genera based on importance according to a random forest analysis; (**D**) a Venn diagram showing seven genera intersecting between importance and relative abundance.

The dominant microbiota at the phylum level in the CON and HF groups were Firmicutes, Bacteroidetes, Spirochaetes, Tenericutes, Proteobacteria, Fibrobacteres, Actinobacteria, TM7, WPS-2, and Verrucomicrobia (relative abundance, *p* > 1%; Figure 2A). Figure 2B shows the top 20 genera in relative abundance at the genus level in the CON and HF groups. Figure 2C shows the top 15 genera ranked by importance, calculated using the random forest algorithm at the genus level, for both groups. Figure 2D shows a Venn diagram of the intersection between the top 20 genera in relative abundance and the top 15 genera in importance at the genus level. As shown in Figure 2D, the overlapping genera were *p-75-a5*, *Lachnospira*, *Roseburia*, *Fibrobacter*, *Phascolarctobacterium*, *Coprococcus*, and *Prevotella*.

The relative abundances of *p-75-a5* (phylum Firmicutes) and *Fibrobacter* were significantly higher in the HF group than in the CON group (*p* < 0.05). No significant differences were observed in the relative abundance of *Lachnospira*, *Roseburia*, *Phascolarctobacterium*, *Coprococcus*, and *Prevotella* between the two groups (*p* > 0.05; Table 8).

**Figure 3:**
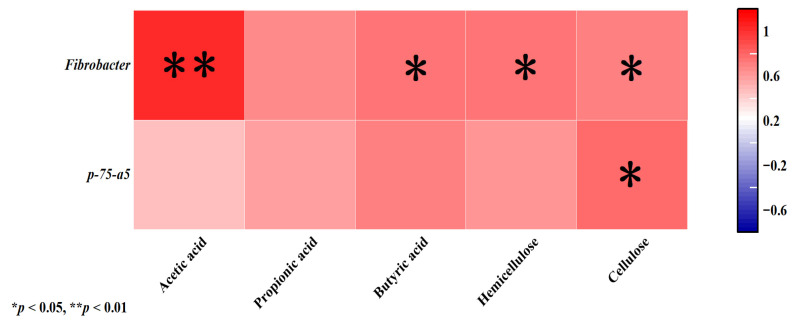
Correlation analysis between the relative abundance of the bacterial community in horizontal feces and short-chain fatty acids, cellulase, and hemicellulase. Red indicates positive correlation; purple indicates negative correlation. “*” indicates a statistically significant correlation between corresponding short-chain fatty acids, enzymes, and bacterial communities (*p <* 0.05). “**” indicates that the correlation between the corresponding short-chain fatty acids, enzymes, and bacterial communities reached a statistically significant level (*p <* 0.01).

*Fibrobacter* showed a highly significant positive correlation with acetic acid (*p*< 0.01) and was significantly associated with butyric acid, cellulase, and hemicellulase (*p* < 0.05). *p-75-a5* exhibited a significant positive correlation with cellulose (*p* < 0.05; Figure 3).

## 4. Discussion

### 4.1. Effects of High-Fiber Diets on the Growth Performance and Health Status of Tibetan Pigs

Growth performance is a fundamental metric for assessing the advantages of animal husbandry and is intricately linked to economic returns. Optimal fiber levels are crucial in enhancing the growth performance and microbial bacterial maturation of pigs [34]. Incorporating wheat bran fiber into the diet of growing pigs within a suitable range does not significantly affect final body weight, average daily gain, or feed efficiency [35]. In one study, it was found that the ADFI and ADG of pigs remained unaffected by the inclusion of wheat straw powder [36]. Similarly, in a different study, altering the levels of wheat bran fiber within the optimal fiber range did not significantly affect the ADFI and ADG of pigs [37]. This indicates that maintaining fiber levels within an appropriate range sustains stable growth performance in pigs without the adverse effects of a high-fiber diet. These findings align with the outcomes of this research, indicating that 10% crude fiber concentration is a suitable range for fiber supplementation in Tibetan pigs under the specific conditions of this study. It should be particularly noted that the addition of 4.9% soybean oil in this study will not affect the conclusions of related research. At this addition level, there is no significant fluctuation in the daily weight gain and feed intake of Tibetan pigs, and studies have shown that an addition of 4% soybean oil can improve the efficiency of pig feed intake without affecting their early growth, neither interfering with development nor masking the effects of fiber [38]. Secondly, studies have confirmed that adding 6% olive oil, sunflower oil, and other vegetable oils to high-concentration diets does not disrupt the overall structural stability of microbial communities, and indicators directly related to bacterial metabolic functions such as substrate degradation efficiency and total volatile fatty acid production show no significant changes [39,40].

Nutrient transformation and utilization within the digestive system of pigs are interconnected processes rather than operating in isolation; they collectively constitute a cohesive system [41]. Apparent digestibility is an indicator of dietary utilization in pigs, as well as their digestive and metabolic conditions [42]. Replacing corn starch with various high-fiber additives in pig feed does not significantly affect the coefficient of total tract apparent digestibility (CTTAD) of nutrients such as dry matter and organic matter across different levels of supplementation [43]. This finding aligns with the results of the present study, which revealed no significant differences in the digestibility of crude protein and ether extract in Tibetan pigs fed diets with varying crude fiber proportions. This indicates that fibers from different sources, due to their varying physicochemical properties, affect the transport speed of digesta in the pig’s gastrointestinal tract and the sites of nutrient digestion [44]. Often, the total fiber digestibility is limited by the indigestible components in the diet, setting an upper limit for digestibility [45]. Therefore, increasing the level of fiber within the digestible range will stimulate the growth of microorganisms capable of utilizing these components and the production of corresponding enzymes. In terms of digestive volume, high-fiber groups have a higher total amount of dietary fiber, but the actual absolute amount of fiber digested differs from the control group. However, due to the presence of indigestible parts, the apparent overall digestibility per unit weight of feed may not show a linear increase. Consequently, substituting low-fiber diets with high-fiber alternatives is feasible.

The blood physiology of Tibetan pigs at high altitudes is intricately linked to their adaptation to hypoxia and disease resistance. Conversely, serum biochemical parameters are associated with animal growth and metabolism and are indicators of nutrient utilization, overall health, and growth performance [46]. The administration of high-fiber diets to pigs does not lead to significant changes in their white and red blood cell counts, liver and kidney functions, glucose and lipid metabolism, and other physiological indicators [47]. This suggests that, provided the fiber content remains within the animal’s tolerance threshold, high-fiber diets do not have any pathological effects on blood and serum parameters [48]. In a separate investigation on Duroc-Landrace-Yorkshire (DLY) pigs, appropriate levels of dietary fiber maintained blood homeostasis without inducing metabolic stress [49,50]. These findings indicate that high-fiber diets maintain overall health and basal metabolism, while concurrently reducing breeding expenses and preserving the normal growth performance of Tibetan pigs. Consequently, this provides a solid foundation for the large-scale breeding of Tibetan pigs.

### 4.2. Response Mechanisms of Bacterial Flora, Enzyme Activities, and Metabolites Under High Fiber Regulation

Cellulose, a product of photosynthesis following the accumulation of significant plant biomass, functions as the primary structural element in plant cell walls [51]. Dietary fiber, in which cellulose plays a crucial role, is a valuable carbohydrate source for the intestinal microbiota. This substrate is an energy reservoir for gut microbiota and a carbon reservoir for the host [52,53]. Animals lack the cellulases necessary to directly digest cellulose [54]. Therefore, cellulose relies on the gut microbiota for breakdown during anaerobic fermentation. Cellulose, a photosynthetic product, is degraded by cellulolytic microorganisms. These microorganisms employ two primary mechanisms: the free cellulase mechanism, in which various secreted enzymes work synergistically [55], and the use of cellulolytic enzyme complexes attached to the outer wall (cellulosomes) for cellulose digestion, resulting in the production of short-chain fatty acids [56]. Short-chain fatty acids, the main microbial metabolites derived from fiber fermentation, play a central role in maintaining intestinal integrity and energy metabolism in Tibetan pigs. *Fibrobacter*, the sole anaerobic Gram-negative bacterium within the *Fibrobacteraceae* family of the *Fibrobacter* order, is characterized by rod-shaped or polymorphic oval cells measuring (3–5 μm × 0.8–1.6 μm). It is recognized as the primary cellulose-degrading bacterium in the gastrointestinal tract of animals [57,58]. In our study, a significant positive correlation was observed between *Fibrobacter* and acetic acid, cellulase, and hemicellulase levels following the administration of high-fiber diets to Tibetan pigs. This research only shows statistical correlations between bacterial genera, SCFAs, and enzymes, and additional experiments are required to verify potential regulatory directions. This correlation can be attributed to *Fibrobacter’s* reliance on carbon dioxide (CO_2_), short-chain fatty acids, ammonia (NH_3_), and multivitamins for growth. *Fibrobacter* exhibits a specific ability to ferment cellulose and cellobiose, while showing a limited capacity to metabolize other sugars. Through the pentose phosphate pathway (PPP) and glycolytic pathway (EMP), *Fibrobacter* converts its substrates into pyruvate when exposed to high-fiber diets, leading to the production of acetic acid and succinate, with occasional minor byproduct formation. These distinctive metabolic characteristics enable *Fibrobacter* to occupy a specialized niche within the intestinal microecology [59,60,61,62,63,64,65]. Acetic acid, a metabolic byproduct, is crucial in regulating intestinal health by providing essential energy to intestinal epithelial cells and facilitating their physiological functions [66]. Additionally, it modulates the intestinal microbiota balance by promoting the growth of beneficial bacteria and inhibiting harmful bacteria proliferation [67]. *Fibrobacter* produces various cellulose-degrading enzymes, including endoglucanases and exoglucanases. The cellulosome structure on the cell wall efficiently anchors the cellulose microfibrils, facilitating the breakdown of cellulose into cellobiose and glucose via the synergistic action of multiple enzymes [68]. The β-1,4 glucanase enzyme of *Fibrobacter* demonstrates optimal activity at a pH range of 5.5–6.5, aligning well with the acidic conditions found in the posterior segment of the porcine intestine. This pH-dependent regulation of enzyme function confers a significant functional advantage for the degradation of dietary fiber [69]. This degradation process provides fermentation substrates for *p-75-a5*, establishing a “substrate relay” mechanism. A diet rich in fiber enhances the synthesis of B vitamins, thereby improving the host’s intestinal immunity [70], and stimulates the growth of *Fibrobacter* through a positive feedback loop.

The genus *p-75-a5*, belonging to the family Erysipelotrichaceae, is a Gram-positive, obligate anaerobic, non-spore-forming, and non-motile bacterium [71]. Its abundance is positively associated with carbohydrate digestion in high-fiber diets [72,73]. As a member of the Erysipelotrichaceae family, *p-75-a5* exhibits functional characteristics that align with the overall metabolic potential of the family. It is a core functional bacterium in animal intestines and is a key contributor to butyric acid production [74]. Butyric acid promotes Interleukin-18 (IL-18) transcription by activating the G Protein-Coupled Receptor 109A (GPR109A) and G Protein-Coupled Receptor 43 (GPR43) receptors and induces the NOD-Like Receptor Pyrin Domain-Containing Protein 3 (NLRP3) inflammasome, thereby reducing mucosal inflammation, enhancing intestinal barrier integrity, and preventing bacterial invasion [75,76,77,78]. Erysipelotrichaceae, a thick-walled phylum, is rich in carbohydrate-active enzyme (CAZyme) genes, particularly cellulase and xylanase, which degrade complex carbohydrates into oligosaccharides and monosaccharides and facilitate subsequent acid production during fermentation [79,80,81,82,83,84]. The Erysipelotrichaceae CAZyme genes in Dansoniaceae are significantly correlated with carbohydrate metabolic pathways, such as glycolysis and amino sugar metabolism [85,86]. As obligate anaerobes, members of the family *Erysipelotrichaceae* convert carbohydrates into pyruvate via glycolysis, ultimately producing acetic and butyric acid [87,88,89].

In this experiment, a high abundance of *Fibrobacter*, *p-75-a5*, and related bacteria was detected in Tibetan pigs. These bacterial groups were positively correlated with the production of dietary cellulase, hemicellulase, and short-chain fatty acids. This finding confirms that Tibetan pigs can efficiently degrade dietary fiber, which is closely linked to the colonization by these fecal microbiota. However, it is necessary to acknowledge certain limitations in this study. First, regarding the effect of animal age, the experiment used 120-day-old growing Tibetan pigs, with a trial duration of 28 days, which only covered their early growth phase, whereas Tibetan pigs typically reach market weight and are slaughtered at 240 to 300 days. It remains to be verified whether the observed synergistic action of “bacteria-enzymes-metabolites” in fiber degradation is stable during their later growth phase. Second, regarding the long-term adaptation mechanism, this study focused on adaptive changes within 28 days to a high-fiber diet, but it cannot fully reflect the inherent long-term adaptive regulatory mechanisms of Tibetan pigs that have evolved on the Tibetan Plateau through natural selection. Finally, it should be noted that the SCFAs and fiber-degrading enzyme activities analyzed in this study were measured in fecal samples, which may not fully represent their production, absorption, or concentration within the intestinal tract. Nevertheless, a 10% crude fiber level complies with animal welfare principles, allowing Tibetan pigs to fully access fibrous feed sources and maintain their inherent characteristics of fiber utilization, laying the foundation for subsequent long-term trials. The practical application of this scheme in live pig production awaits further verification. This finding fills the theoretical gap for high-fiber standards in large-scale confined Tibetan pigs, providing a theoretical basis for developing specialized feed, reducing costs through low-cost high-fiber raw materials, and ensuring the healthy growth of large-scale farmed Tibetan pigs.

## 5. Conclusions

Our findings provide strong evidence that the effective utilization of dietary fiber by Tibetan pigs results from highly specialized microbial mechanisms in the large intestine, as reflected by their fecal microbiota composition. *Fibrobacter* and *p-75-a5* are key to effective fiber utilization in these pigs. Specific microorganisms secreted more enzymes to promote dietary fiber breakdown, resulting in more metabolites. This study may inspire new ways to deepen the understanding of physiological traits in regional species, improve dietary fiber, and fully exploit fiber adaptation in Tibetan pigs.

## Figures and Tables

**Table 1:** Dietary compositions and chemical component contents (air dry matter basis, %).

Item	CON	HF
Ingredients (%)	-	-
Corn	67.22	52.35
Wheat Middling	9.000	9.000
Soybean Oil	-	4.900
Wood Cellulose	-	8.200
Soybean Meal	10.00	10.00
Soybean Protein Concentrate	5.000	6.900
Soybean Hulls	6.000	6.000
CaCO_3_	0.850	0.880
CaHPO_4_	0.360	0.350
L-Lysine hydrochloride	0.245	0.150
DL-Threonine	0.075	0.040
Tryptophan	0.020	-
Salt	0.200	0.200
Choline	0.100	0.100
Premix ^1^	0.130	0.130
Diatomite	0.800	0.800
Chemical Components ^3^	-	-
Digestible Energy (MJ/kg)	13.89	13.89
Crude Protein	15.00	15.00
Crude Fiber	5.000	10.00
Calcium	0.600	0.600
Available Phosphorus	0.380	0.360
L-Lysine	0.750	0.750
Methionine + Cysteine	0.430	0.430
Threonine	0.500	0.500
Tryptophan	0.140	0.140
Acid-insoluble ash	0.963	1.109

Abbreviations: CON, the diet contains 5% crude fiber; HF, the diet contains 10% crude fiber; ^1^. Premix provided per kilogram of diet: Vitamin A 1700 IU, Vitamin D_3_ 200 IU, Vitamin E 15 mg, Vitamin K 0.5 mg, Thiamin 1.5 mg, Riboflavin 3 mg, Nincin 15 mg, Pantothenic acid 10 mg, Pyridoxine 1.5 mg, Folic acid 0.4 mg, Biotin 0.08 mg, Vitamin B_12_ 15 mg, Fe 70 mg, Cu 4.5 mg, Mn 4 mg, Zn 75 mg, I 0.15 mg, Se 0.25 mg. ^3^. Soybean oil was added to the HF group to adjust digestible energy to match the CON group.

**Table 2:** Effects of a high-fiber diet on the growth performance of Tibetan pigs.

Item	CON	HF	SEM	*p*-Value
1–2 weeks				
IBW (kg)	21.52	20.48	0.590	0.390
FBW (kg)	27.32	26.10	0.650	0.350
ADG (g/d)	386.8	374.7	10.47	0.570
ADFI (g/d)	1296	1178	38.63	0.130
F:G	3.350	3.240	0.100	0.600
3–4 weeks				
FBW (kg)	32.88	31.64	0.73	0.410
ADG (g/d)	397.3	381.1	25.92	0.770
ADFI (g/d)	1539	1440	46.35	0.290
F:G	3.940	3.820	0.450	0.350
1–4 weeks				
ADG (g/d)	392.1	393.9	12.10	0.950
ADFI (g/d)	1418	1309	40.70	0.190
F:G	3.630	3.490	0.130	0.620

Abbreviations: CON, the diet contains 5% crude fiber; HF, the diet contains 10% crude fiber; IBW, initial body weight; FBW, final body weight; ADG, average daily gain; ADFI, average daily feed intake; F:G, feed/gain ratio. Values are means ± pooled SEM (*n* = 40).

**Table 3:** Effects of a high-fiber diet on the apparent nutrient digestibility of Tibetan pigs.

Item	CON	HF	SEM	*p*-Value
CP (%)	78.53	78.69	0.39	0.84
EE (%)	84.92	85.38	0.38	0.56
CF (%)	63.28	63.40	0.87	0.95

Abbreviations: CON, the diet contains 5% crude fiber; HF, the diet contains 10% crude fiber; CP, crude protein; EE, ether extract; CF, crude fiber. Values are means ± pooled SEM (*n* = 40).

**Table 4:** Effects of a high-fiber diet on the blood parameters of Tibetan pigs.

Item	CON	HF	SEM	*p*-Value
WBC (10^9^/L)	21.84	21.86	0.960	0.992
RBC (10^12^/L)	9.210	8.880	0.150	0.291
HGB (g/L)	161.8	156.3	3.140	0.395
HCT (%)	50.04	47.80	1.060	0.305
MCV (fL)	54.30	53.93	0.600	0.767
MCH (g/L)	17.51	17.56	0.190	0.899
MCHC (g/L)	323.5	326.6	1.200	0.206
RDW (%)	17.14	16.84	0.34	0.667
PLT (10^9^/L)	429.4	393.4	10.15	0.075
MPV (fL)	7.010	7.310	0.130	0.260
PDW (%)	16.31	16.42	0.070	0.452
PCT (%)	0.310	0.270	0.010	0.087

Abbreviations: CON, the diet contains 5% crude fiber; HF, the diet contains 10% crude fiber; WBC, white blood cells; RBC, red blood cells; HGB, hemoglobin; HCT, hematocrit; MCV, mean red blood cell volume; MCH, mean corpuscular hemoglobin content; MCHC, mean corpuscular hemoglobin concentration; RDW, red blood cell distribution width; PLT, platelets; MPV, mean platelet volume; PDW, platelet distribution width; PCT, procalcitonin. Values are means ± merged SEM (*n* = 40).

**Table 5:** Effects of a high-fiber diet on the serum parameters of Tibetan pigs.

Item	CON	HF	SEM	*p*-Value
ALT (U/L)	46.46	47.64	2.260	0.801
AST (U/L)	35.12	34.16	1.760	0.794
AST/ALT	1.080	0.720	0.160	0.261
TP (g/L)	72.88	70.05	1.150	0.228
ALB (g/L)	39.76	36.79	0.880	0.092
GLO (g/L)	33.11	33.26	0.910	0.937
A/G	1.220	1.140	0.040	0.318
ALP (U/L)	207.0	176.8	14.68	0.317
UREA (mmol/L)	5.060	5.080	0.340	0.987
CREA (μmol/L)	65.13	61.20	2.860	0.506
TC (mmol/L)	2.790	2.580	0.110	0.373
GlU (mmol/L)	4.880	4.960	0.160	0.806

Abbreviations: CON, the diet contains 5% crude fiber; HF, the diet contains 10% crude fiber; ALT, alanine aminotransferase; AST, aspartate aminotransferase/alanine aminotransferase ratio; AST/ALT, grain-to-grass ratio; TP, total protein; ALB, albumin; GLO, globulin; A/G, albumin/globulin ratio; ALP, serum alkaline phosphatase assay; UREA, urea; CREA, creatinine; TC, total cholesterol; GlU, glucose measurement. Values are the means ± merged SEM (*n* = 40).

**Table 6:** Effects of a high-fiber diet on the enzyme activity of Tibetan pigs.

Item	CON	HF	SEM	*p*-Value
Cellulase (IU/L)	311.1	341.5	7.630	0.040
Hemicellulase (IU/L)	295.3	348.7	10.16	0.030

Abbreviations: CON, the diet contains 5% crude fiber; HF, the diet contains 10% crude fiber; Values are the means ± merged SEM (*n* = 40).

**Table 7:** Effects of a high-fiber diet on SCFAs of Tibetan pigs.

Item	CON	HF	SEM	*p*-Value
Acetic acid (mg/g)	2156	3068	153.0	0.020
Formic acid (mg/g)	1360	1418	97.13	0.770
Propionic acid (mg/g)	100.6	147.5	15.06	0.040
Isobutyric acid (mg/g)	473.7	482.6	20.46	0.840
Butyric acid (mg/g)	110.2	194.5	23.92	0.020
Isovaleric acid (mg/g)	133.0	129.0	7.330	0.800
Valeric acid (mg/g)	36.02	37.54	1.860	0.700

Abbreviations: CON, the diet contains 5% crude fiber; HF, the diet contains 10% crude fiber; Values are the means ± merged SEM (*n* = 40).

**Table 8:** Differences in the relative abundances of the cross-genera between the two groups (%).

Item	CON	HF	SEM	*p*-Value
*Prevotella*	7.690	5.920	0.570	0.130
*Phascolarctobacterium*	4.390	3.110	0.400	0.120
*Coprococcus*	0.820	0.580	0.130	0.400
*Fibrobacter*	0.180	0.990	0.160	0.020
*p-75-a5*	0.230	0.790	0.100	0.020
*Roseburia*	0.250	0.240	0.050	0.960
*Lachnospira*	0.510	0.370	0.910	0.480

Abbreviations: CON, the diet contains 5% crude fiber; HF, the diet contains 10% crude fiber; Values are the means ± merged SEM (*n* = 20).

## Data Availability

The original contributions presented in this study are included on FigShare at. DOI: 10.6084/m9.figshare.30225790.
